# Health-related selection into employment among the unemployed

**DOI:** 10.1186/s12889-022-13023-0

**Published:** 2022-04-05

**Authors:** Liina Junna, Heta Moustgaard, Pekka Martikainen

**Affiliations:** 1 Population Research Unit, University of Helsinki, Unioninkatu 35 (P.O. Box 18), 00014 Helsinki, Finland; 2 Max Planck Institute for Demographic Research, Konrad-Zuse-Straße 1, 18057 Rostock, Germany; 3 Helsinki Institute for Social Sciences and Humanities, University of Helsinki, Vuorikatu 3, 00014 Helsinki, Finland; 4 Centre for Health Equity Studies, University of Stockholm and Karolinska Institute, Stockholm University, SE-106 91 Stockholm, Sweden

**Keywords:** Re-employment, Employment, Unemployment, Longitudinal study, Health

## Abstract

**Background:**

Successful transitions from unemployment to employment are an important concern, yet little is known about health-related selection into employment. We assessed the association of various physical and psychiatric conditions with finding employment, and employment stability.

**Methods:**

Using total population register data, we followed Finnish residents aged 30–60 with an unemployment spell during 2009–2018 (*n* = 814,085) for two years from the onset of unemployment. We predicted any, stable, and unstable employment by health status using Cox proportional hazards models. The data on specialized health care and prescription reimbursement were used to identify any alcohol-related conditions and poisonings, psychiatric conditions and self-harm, injuries, and physical conditions. We further separated physical conditions into cancer, diabetes, heart disease, and neurological conditions, and psychiatric conditions into depression, anxiety disorders and substance use disorders.

**Results:**

The likelihood of any employment was lower among those who had any of the assessed health conditions. It was lowest among those with alcohol-related or psychiatric conditions with an age-adjusted hazard ratio of 0.45 (95% confidence interval 0.44, 0.46) among men and 0.39 (0.38, 0.41) among women for alcohol-related and 0.64 (0.63, 0.65) and 0.66 (0.65, 0.67) for psychiatric conditions, respectively. These results were not driven by differences in socioeconomic characteristics or comorbidities. All the included conditions were detrimental to both stable and unstable employment, however alcohol-related and psychiatric conditions were more harmful for stable than for unstable employment.

**Conclusions:**

The prospects of the unemployed finding employment are reduced by poor health, particularly alcohol-related and psychiatric conditions. These two conditions may also lead to unstable career trajectories. The selection process contributes to the health differentials between employed and unemployed people. Unemployed people with health problems may therefore need additional support to improve their chances of employment.

**Supplementary Information:**

The online version contains supplementary material available at 10.1186/s12889-022-13023-0.

## Background

The unemployed have poorer health when compared to the employed population [1–3], but previous research has predominantly focused on whether this association is causal from unemployment to health. The association between unemployment and poor health could also be due to health-related selection, which refers to the hypothesis that poor health – through various mechanisms – increases the risk of unemployment [4].

Health-related selection may function through both an increased likelihood of job loss and a decreased likelihood of finding employment once unemployed. As for the former, poor health may negatively influence work performance (e.g., productivity, social relations) or increase workplace absences, causing an increased risk of being laid off [5]. Some empirical evidence suggests that poor mental health and substance-use problems are associated with an increased risk of job loss, while the evidence concerning physical health is weaker [6–13].

However, few studies have assessed the role of health in finding employment once unemployed. There are various reasons to expect that unemployed people with poor health may have difficulties in securing employment. Health is likely to affect employment through human capital formation: Those with poor health may have attained lower educational or skill levels, which in turn reflects on their likelihood of finding employment, and in particular, good quality, stable employment [14, 15]. Unemployed job seekers with health conditions may also narrow their job search to positions with low perceived health-related demands (e.g., lower physical demands, less stress or competition), which further limits their options [16–19]. Job searches may be hindered by symptoms, fatigue, or health-related stress, although there is a lack of empirical evidence to assess the importance of these factors [16–19]. Some of the unemployed also have limited capacity for work and may either remain home to focus on unpaid labor, or they wait to exit from the labor force (e.g. while their disability pension awaits approval) [17, 20].

Both the behavior of the applicant and discrimination by the employer may contribute to health-related selection. Studies have demonstrated that employers view signs of poor health, such as visible symptoms or gaps in employment history, to imply risk (e.g., sickness absences, poor productivity) [21, 22], or associate them with other unwanted applicant characteristics (e.g., poor emotional or social skills) [16, 17, 23–25]. However, employers are unlikely to possess full knowledge of an applicant’s health. As different psychiatric and physical conditions display different visible cues, this may impact hiring decisions differently. Certain cues may also be more associated with unwanted characteristics than others. Studies report examples of such prejudices towards the obese [23, 25] and those with mental health problems [25]. Although little is known about the relative importance of physical and psychiatric health, some evidence suggests that those with alcohol-related and psychiatric problems may be more discriminated against in comparison to those with physical conditions [25, 26].

The majority of the empirical studies conducted on the role of health in finding employment have addressed general health measures such as self-rated health. Among the Dutch unemployed, poor self-rated health was associated with a lower likelihood of returning to work [27]. In Australia, similar results were reported for long-standing health conditions [28]. Furthermore, during an economic downturn in Scandinavia, those with poor self-rated health had a lower probability of securing new employment in Sweden but not in Denmark or Norway, while long-standing illnesses were a disadvantage in Norway but not in Sweden or Denmark [29]. Thus, the association between general self-reported health and employment appears somewhat inconsistent across health measures and country contexts [30, 31].

Using general health measures also makes it difficult to establish which aspects of health underlie health-related selection into employment, and even less is known about specific health conditions. According to the scant evidence, mental health may be particularly relevant. Among the long-term unemployed in Norway, any psychiatric diagnosis identified in a clinical examination was associated with a considerably lower likelihood of finding employment [7, 8], while in Denmark, the same applies to purchasing any psychiatric medications [32]. Two studies found no association for alcohol use disorders [9] and distress [5].

Even fewer studies have explored the relation between physical health and employment. The purchase of prescription medication for any physical condition has been demonstrated to lower the likelihood of employment [32]. A study series in Norway addressed any diagnoses related to physical conditions received during a medical examination, but the numbers of study participants were small and the results indeterminate (e.g., 5-year employment OR 0.60; 95% CI: 0.30, 1.18; 31 unemployed subjects with a physical condition) [7, 8]. Some evidence also suggests that physical symptoms could hinder a person’s employment. This was reported in Sweden for circulatory and general symptoms, (for both, β − 0.17; standard error SE 0.08) [10] and for lower back pain (OR 0.37; 95% CI: 0.15, 0.92) and pain in the lower extremities in Finland (OR 0.38; 95% CI: 0.15, 0.93) [33], while in Norway, symptoms were unassociated with finding employment [7].

To summarize, the current literature has various limitations and gaps concerning health-related selection from unemployment into employment. The majority of previous studies have relied on small samples (*n* = 210–1856) and provide only tentative evidence. To date, few large-scale, general population studies have compared different aspects of health, such as physical and mental health. There is even less published data on how specific physical and mental health conditions affect finding employment. There is also little current data on health and employment stability [34] even though stability is particularly important, given that procuring stable, good quality employment is associated with positive changes in health [35]. The question of health-related selection from unemployment to employment is thus important both from the perspective of an individual, public health and finance. Moreover, health-related selection from unemployment to employment also adds a crucial, and largely missing, piece to the general discussion on the mechanisms that link unemployment with poor health.

Our study makes three main contributions. First, we estimate the magnitude of health-related selection into employment among unemployed men and women across various aspects of health from physical to psychiatric as well as for specific health conditions. Second, we also address employment stability. Third, we analyze large general population register data (*n* = 814,085) that does not suffer from the problems associated with survey studies, such as selective attrition and non-response. Furthermore, we adjust for various individual characteristics (e.g., education, age, and employment history) as well as the labor market context, which are all likely to affect employment.

Our research questions are as follows:

1. Do different general aspects of health (any alcohol-related conditions and poisonings, any psychiatric conditions and self-harm, any injuries, and any physical conditions) predict finding employment among unemployed men and women?
2. Do specific physical conditions (cancer, diabetes, heart disease, neurological conditions) and psychiatric health conditions (depression, anxiety disorders, substance use disorders) predict finding employment among unemployed men and women?
3. Do the different general aspects of health and the specific health conditions predict finding a) stable employment and b) unstable employment?

## Methods

### The data

**Fig. 1:**
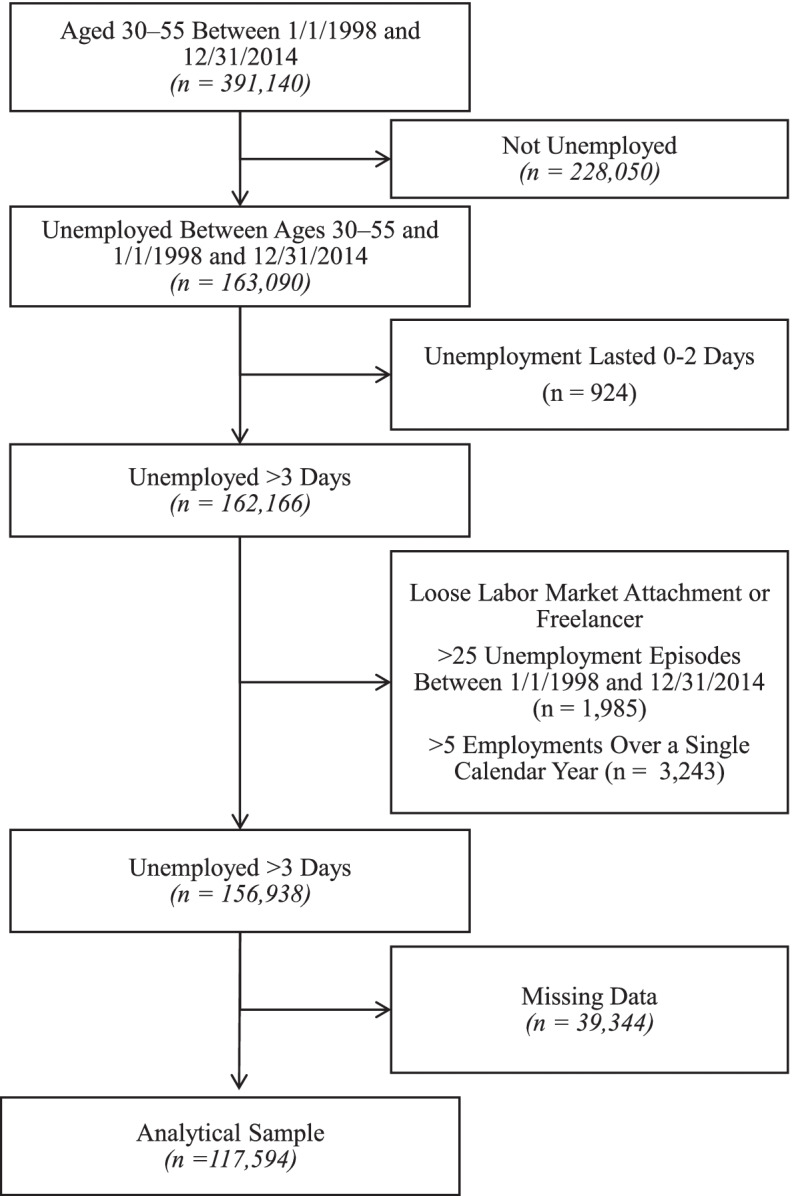
Flow chart of those included in the analytical sample, unemployed Finnish residents aged 30**–**60 between the years 2009 and 2018

We analyzed the total population of unemployed Finnish residents aged 30**–**60 during the years 2009 to 2018 identified from the population registers of Statistic Finland (Fig. 1; permission #TK-53-339-13). This age range was selected because a substantial share of Finnish residents under the age of 30 are still studying, while individuals above the age of 60 are near retirement age [36]. We linked information from various administrative registers using the personal identification codes that all residents receive at birth or upon immigration.

Unemployment was defined as having registered as currently jobless and seeking employment (*n* = 886,050, Fig. 1). This measure is highly sensitive, as registering immediately at the start of unemployment is required to receive unemployment benefits in Finland. We utilized data on exact unemployment onset and ending dates. The date of registering was set as the onset of unemployment. The end of unemployment was set at the date of registering an exit from unemployment, the end of the two-year follow-up, death or aging out, whichever came first (Table 1). In this analysis, we refer to an unemployment spell as the time in days that someone is registered as unemployed jobseeker. As it is possible to experience multiple spells of unemployment, we considered all spells from each individual (see Statistical Analyses).

**Table 1:** Analytical sample characteristics, Finnish unemployed men and women aged 30–60 in 2009–2018

	Men			Women		
	N	%	Mean [SD]	N	%	Mean [SD]
Individuals	394,999			419,086		
Unemployment spells	1,349,975		4.1 [4.7]	1,508,548		4.8 [6.5]
Unemployment spell duration, days			198.0 [211.1]			161.0 [188.7]
Unemployment spell outcomes						
Unemployed	469,915	34.8		425,128	28.2	
Employed	606,589	44.9		773,739	51.3	
Stable	314,956	23.3		470,717	31.2	
Unstable	291,633	21.6		303,022	20.1	
Training, education, labor market program	264,565	19.6		305,235	20.2	
Migrated, retired, died	8906	0.7		4446	0.3	
Age^a^			44.1 [9.3]			43.9 [9.1]
Education^a^						
Basic		54.0			46.0	
Secondary		49.9			50.1	
Tertiary		35.6			64.4	
Health conditions prior to unemployment onset^a^						
Any alcohol-related conditions, poisonings	62,084	4.6		23,297	1.5	
Any psychiatric conditions, self-harm	61,186	4.5		99,996	6.6	
Depression	35,803	2.7		58.259	3.9	
Anxiety disorders	16,315	1.2		23,932	1.6	
Substance use disorders	57,223	4.2		21,095	1.4	
Any injuries	157,716	11.7		116,117	7.7	
Any physical conditions	401,426	29.7		559,999	37.1	
Cancer	11,334	0.8		25,633	1.7	
Diabetes	62,357	4.6		46,505	3.1	
Heart disease	45,829	3.4		25,770	1.7	
Neurological conditions	67,711	5.0		82,889	5.5	

Abbreviations: *SD* standard deviation

^a^ Presented as the share of all unemployment spells. Stable employment was defined as still being employed at the end of the calendar year following the initial date of employment, and unstable employment as no longer being employed. For the definitions of the general aspects of health and the specific health conditions, please see Measurements and Additional file 2

We excluded spells lasting two days or less and ending in employment, as these are likely to represent workplace transitions (Fig. 1). We further excluded recurrent unemployment spells resulting in employment (five or more spells over a calendar year), as these are likely to be freelancers in between jobs. We also entirely excluded individuals who were considered to be weakly attached to the labor market (those with over 25 separate unemployment spells during the study period). Including these individuals in a sensitivity analysis had a minimal impact on our results (Supplementary Table 1, 2; Additional file 1). We excluded unemployment spells that had missing data on end dates, sociodemographic measures, or health status. Data were missing for less than 0.8% of all unemployment episodes and therefore this exclusion is unlikely to greatly affect our results. Furthermore, individuals with missing data for an unemployment spell could nevertheless enter the analytical sample if they had another unemployment spell for which these measures were available. In addition, individuals who only had unemployment spells with missing background data were excluded entirely; this constituted a total of 66,847 individuals (Fig. 1). Most of these cases consist of very recent immigrants or return migrants who only had one unemployment spell immediately after entering the Finnish registers, which meant that measures on health or other individual characteristics prior to the onset of unemployment could not be determined. The final analytical sample consisted of 814,085 individuals, with a total of 2,858,523 unemployment spells.

### Measures

The outcome was the exact date of exit from unemployment due to finding employment. We also separately analyzed stable and unstable employment (i.e., if the unemployed with poor health opt for, or can only obtain, insecure or short-term jobs). Stable employment was defined as being employed at any workplace at the end of the calendar year following the initial date of employment. Unstable employment was defined as not being employed at the end of the calendar year following employment.

The exposure was the pre-baseline health status measured over the two years preceding the onset of unemployment; the information on health status and other covariates was therefore recorded in 2007–2019. All included health measures were based on information on visits to specialized health care from the Care Register for Health Care of the National Institute for Health and Welfare (hospitalizations, outpatient care, emergency care visits). For the specific physical conditions only, we additionally used information on special reimbursement rights for long-term medication for certain chronic diseases (the right to full or nearly full financial compensation upon purchase of medication), obtained from the registers of the Social Insurance Institution of Finland (Additional file 2). To obtain the right to special reimbursement, patients must provide a clinician’s confirmation of their need for the prescription due to a specific diagnosis [37]. The special reimbursement system covers medications that are primarily used to treat certain severe physical conditions and cannot be used to identify common mental health conditions, or physical conditions outside this reimbursement system. The care and medication registers are collected for administrative purposes of the publicly funded universal health insurance and cover all Finnish residents. They include the dates (visits to specialized care/beginning of special reimbursement right) and the main diagnoses (International Classification of Diseases, revision 10 (ICD-10)/Finnish special refund category).

The selection of the health conditions was based on their high prevalence and public health importance among working-age Finns [38]. First, we explored different general aspects of health. We considered visits to specialized care that were related to 1) any alcohol-related conditions and poisonings; 2) any psychiatric conditions and self-harm (excluding alcohol and other substance use disorders); 3) any injuries; and 4) any physical conditions (excluding visits related to pregnancy and childbirth, and alcohol-related physical conditions, see Additional file 2). We then separately considered common specific health conditions. Using data on both specialized health care visits and special reimbursement rights, we separated the physical conditions as follows: 1) cancer; 2) diabetes; 3) heart disease; and 4) neurological conditions. Based on specialized health-care visit data, we divided the psychiatric conditions into 1) depression; 2) anxiety disorders; and 3) substance use disorders. All health indicators – both the general aspects of health and the specific health conditions – were included as separate dummy measures of whether the individual had the given condition over the two calendar years preceding the onset year of unemployment (yes/no).

Based on previous literature [1, 11, 13], we controlled for the following additional characteristics: continuous age, native language (Finnish or Swedish, other), marital status (married or cohabiting, single, divorced or widowed), education (basic, secondary, or tertiary), occupational status (upper or lower-level white collar with managerial occupations, lower-level white collar, blue collar, farmer or self-employed, student, or other) [39], number of individuals living in the same household (continuous), home ownership (yes/no), unemployment history (number of months unemployed over the two calendar years preceding the onset of unemployment, continuous), running number of the unemployment spells (from the start of the study period, categorical), and the onset year of unemployment (categorical). As labor market prospects may be particularly limited in declining regions, we also included the region of residence (Nomenclature of Territorial Units for Statistics 3, categorical, 21 regions). All these covariates were measured annually on December 31 of the year preceding the onset of unemployment and included as time-invariant. For the analyses concerning the specific health conditions, we also generated a binary measure for any remaining comorbidity other than the specific health conditions considered in this study (any visits to health care over the preceding two years for any diagnosis excluding cancer, diabetes, heart disease, neurological conditions, depression, anxiety disorders, substance use disorders; yes/no). For women, we additionally controlled for births that occurred over the two preceding years (yes/no), which were identified from the hospital discharge register, as having a child may affect the risk and length of unemployment.

### Statistical analyses

We conducted multivariate Cox proportional hazards models in Stata, version 16.1 (College Station, Texas, 2019). The analysis time was defined as the number of days from the onset of unemployment. As an individual could experience unemployment and find employment multiple times, we considered all spells for each individual, with standard errors clustered at the individual level. There was no loss to follow-up. Individuals were right-censored at the end of their unemployment spell (which could end due to employment, retirement, migration, exiting the labor market for other reasons, aging out, or death), or the end of the two-year follow-up, whichever came first. The proportional hazards assumption was visually estimated to hold well for all outcomes (results upon request from the authors).

We stratified all analyses by sex because health may have a gendered impact for finding employment, and because less is known about the employment among unemployed women [40]. We separately estimated the following models for the four general aspects of health, and the seven specific health conditions (exposure, see above). In Model 1, we included each health measure separately and controlled for age and the unemployment onset year. In Model 2, we added the other individual characteristics (see other covariates, above). In Model 3a, we considered all the general aspects of health simultaneously to control for the effects of comorbidity while controlling for the other covariates as in Model 2. In Model 3b, all the specific health condition were considered together and, in addition to the covariates introduced in Model 2, we further adjusted for a binary measure for any remaining comorbidity (see the other covariates above). We also separately considered the role of the different general aspects of health and the specific conditions for finding stable and unstable employment, while adjusting for the same variables as in Models 3a and 3b.

### Sensitivity analyses

Some of the brief unemployment spells could reflect transitions from one workplace to another rather than involuntary unemployment. To address this, we excluded unemployment episodes from the analyses that lasted less than a month (Supplementary Table 1, 2; Additional file 1). As our exclusion criteria were arbitrary for weak labor market attachment and freelancing (over 25 unemployment spells, or more than 5 spells ending in employment over a calendar year), we also tested the inclusion of these spells (Supplementary Table 1, 2; Additional file 1) [41].

To ensure that our results were not due to competing risks such as retirement and death, we additionally conducted the Fine and Gray competing risk regression for finding any, stable, and unstable employment (Supplementary Table 3; Additional file 1) [36].

## Results

During the two-year follow-up, 48.3% of the unemployed found employment (Table 1). The average length of the follow-up was 280 days among men (standard deviation SD 270) and 219 days among women (SD 253). The median among men was 116 days and the interquartile range was 42–272 days, while among women, the respective figures were 84 days and 32–210 days.

Of the different aspects of health, the conditions that were particularly detrimental to finding any employment were any alcohol-related and any psychiatric conditions: In the age and onset year-adjusted Model 1, alcohol-related conditions more than halved the likelihood of employment (Table 2). Any injuries also lowered the likelihood of employment by approximately one fifth (HR 0.82; 95% CI: 0.81, 0.83 among men and HR 0.81; 95% CI: 0.80, 0.82 among women) and any other physical conditions by slightly over 10% (HR 0.88; 95% CI: 0.88, 0.89 among both men and women). These associations were not greatly attenuated by adjusting for the individual demographic and socioeconomic characteristics and comorbidities in Models 2 and 3. In the fully adjusted Model 3, alcohol-related conditions were still associated with a 53% (95% CI: 0.52, 0.55) reduction in employment among men and a 47% (95% CI: 0.46, 0.49) reduction among women.

**Table 2:** The association between different general aspects of health and finding any employment among unemployed Finnish men (*n* = 394,999) and women (*n* = 419,086) aged 30–60 in 2009–2018^a^

	Model 1^a^		Model 2^b^		Model 3a^c^	
	HR	95% CI	HR	95% CI	HR	95% CI
Men						
Any alcohol-related conditions, poisonings	0.45	(0.44,0.46)	0.52	(0.51,0.53)	0.53	(0.52,0.55)
Any psychiatric conditions, self-harm	0.64	(0.63,0.65)	0.69	(0.67,0.70)	0.69	(0.67,0.70)
Any injuries	0.82	(0.81,0.83)	0.85	(0.84,0.86)	0.92	(0.91,0.93)
Any physical conditions	0.89	(0.88,0.89)	0.88	(0.87,0.89)	0.90	(0.90,0.91)
Women						
Any alcohol-related conditions, poisonings	0.39	(0.38, 0.41)	0.47	(0.45,0.48)	0.47	(0.46,0.49)
Any psychiatric conditions, self-harm	0.66	(0.65,0.67)	0.70	(0.69,0.71)	0.71	(0.70,0.72)
Any injuries	0.81	(0.80,0.82)	0.85	(0.84,0.86)	0.91	(0.90,0.92)
Any physical conditions	0.89	(0.88,0.89)	0.89	(0.88,0.89)	0.90	(0.90,0.91)

Abbreviations: *HR* hazard ratio, *CI* confidence interval

^a^ Analyses conducted using multivariate Cox proportional hazards models; all *p*-values <.001. For the definitions of the general aspects of health, please see Measurements and Additional file 2

^b^ Model 1: Adjusted for age and unemployment onset year; each general aspect of health (any alcohol-related conditions, poisonings; any psychiatric conditions, self-harm; any injuries; any physical conditions) modelled separately

^c^ Model 2: Model 1 + native language, marital status, education, occupational status, number of individuals living in the same household, home ownership, region of residence, unemployment history, number of the unemployment spell, and for women, births over the two preceding years; each general aspect of health (any alcohol-related conditions, poisonings; any psychiatric conditions, self-harm; any injuries; any physical conditions) modelled separately

^d^ Model 3a: Model 2 + all general aspects of health (any alcohol-related conditions, poisonings; any psychiatric conditions, self-harm; any injuries; any physical conditions) modelled simultaneously

All specific physical and psychiatric conditions were also negatively associated with finding any employment among both men and women (Table 3). For example, after adjusting for the other general aspects of health and all covariates, men with diabetes were 15% (95% CI: 0.83, 0.87) less likely and the respective women were 13% (95% CI: 0.81, 0.85) less likely to find employment compared to their healthy counterparts (Table 3, Model 3). The specific psychiatric conditions also lowered the likelihood of finding employment and substance use disorders were particularly harmful.

**Table 3:** The association between specific health conditions and finding any employment among unemployed Finnish men (*n* = 394,999) and women (*n* = 419,086) aged 30–60 in 2009–2018^a^

	Model 1^a^		Model 2^b^		Model 3b^c^	
	HR	95% CI	HR	95% CI	HR	95% CI
Men						
Cancer	0.91	(0.87,0.95)	0.90	(0.87,0.94)	0.90	(0.86,0.93)
Diabetes	0.85	(0.83,0.86)	0.85	(0.83,0.87)	0.85	(0.83,0.87)
Heart disease	0.87	(0.85,0.89)	0.86	(0.84,0.88)	0.87	(0.85,0.89)
Neurological conditions	0.84	(0.83,0.86)	0.81	(0.79,0.83)	0.82	(0.80,0.84)
Depression	0.60	(0.59,0.62)	0.64	(0.63,0.66)	0.70	(0.68,0.72)
Anxiety disorders	0.57	(0.55,0.59)	0.63	(0.61,0.66)	0.74	(0.71,0.76)
Substance use disorders	0.44	(0.43,0.46)	0.52	(0.50,0.53)	0.53	(0.51,0.54)
Women						
Cancer	0.91	(0.88,0.93)	0.89	(0.87,0.92)	0.87	(0.85,0.90)
Diabetes	0.84	(0.82,0.86)	0.84	(0.82,0.86)	0.83	(0.81,0.85)
Heart disease	0.87	(0.85,0.90)	0.86	(0.84,0.89)	0.86	(0.84,0.88)
Neurological conditions	0.85	(0.84,0.86)	0.83	(0.81,0.84)	0.82	(0.81,0.84)
Depression	0.62	(0.61,0.63)	0.66	(0.64,0.67)	0.68	(0.67,0.70)
Anxiety disorders	0.63	(0.61,0.65)	0.68	(0.66,0.70)	0.77	(0.75,0.79)
Substance use disorders	0.39	(0.37,0.40)	0.46	(0.44,0.48)	0.48	(0.46,0.50)

Abbreviations: *HR* hazard ratio, *CI* confidence interval

^a^ Analyses conducted using multivariate Cox proportional hazards models; all p-values <.001. For the definitions of the specific health conditions, please see Measurements and Additional file 2

^b^ Model 1: Adjusted for age and unemployment onset year; each specific health condition (cancer, diabetes, heart disease, neurological conditions, depression, anxiety disorders, substance use disorders) modelled separately

^c^ Model 2: Model 1 + native language, marital status, education, occupational status, number of individuals living in the same household, home ownership, region of residence, unemployment history, number of unemployment spells, remaining comorbidities and for women, births over the two preceding years; each specific health condition (cancer, diabetes, heart disease, neurological conditions, depression, anxiety disorders, substance use disorders) modelled separately

^d^ Model 3b: Model 2 + binary measure for any remaining comorbidity; all specific health conditions modelled simultaneously

The likelihood of finding stable and unstable employment was reduced by any alcohol-related and any psychiatric conditions and to a lesser extent, by injuries and physical conditions (Table 4). Alcohol-related and psychiatric conditions were particularly detrimental to stable employment. Accordingly, the specific physical conditions were associated with both stable and unstable employment, while depression, anxiety disorders and most notably substance use disorders were more strongly associated with stable employment (Table 5).

**Table 4:** The association between different general aspects of health and finding stable and unstable employment among unemployed Finnish men (*n* = 394,999) and women (*n* = 419,086) aged 30–60 in 2009–2018^a^

	Stable		Unstable	
	HR	95% CI	HR	95% CI
Men				
Any alcohol-related conditions, poisonings	0.39	(0.37,0.40)	0.65	(0.63,0.67)
Any psychiatric conditions, self-harm	0.60	(0.59,0.62)	0.80	(0.78,0.82)
Any injuries	0.92	(0.91,0.94)	0.92	(0.90,0.93)
Any physical conditions	0.91	(0.90,0.92)	0.90	(0.89,0.91)
Women				
Any alcohol-related conditions, poisonings	0.36	(0.33,0.38)	0.60	(0.57,0.63)
Any psychiatric conditions, self-harm	0.64	(0.63,0.65)	0.82	(0.81,0.84)
Any injuries	0.90	(0.88,0.91)	0.92	(0.91,0.94)
Any physical conditions	0.90	(0.89,0.91)	0.92	(0.91,0.93)

Abbreviations: *HR* hazard ratio, *CI* confidence interval

^a^ All *p*-values <.001. Model adjusted for age, onset year of unemployment, native language, marital status, education, occupational status, number of individuals living in the same household, home ownership, region of residence, unemployment history, number of the unemployment spell, and for women, births over the two preceding years. All general aspects of health measures modelled simultaneously. Stable employment was defined as still being employed at the end of the calendar year following the initial date of employment, and unstable employment no longer being employed. For the definitions of the general aspects of health measures, please see Measures and Additional file 2

**Table 5:** The association between specific health conditions and finding stable and unstable employment among unemployed Finnish men (*n* = 394,999) and women (*n* = 419,086) aged 30–60 in 2009–2018^a^

	Stable		Unstable	
	HR	95% CI	HR	95% CI
Men				
Cancer	0.88***	(0.84,0.93)	0.91**	(0.86,0.96)
Diabetes	0.82***	(0.80,0.84)	0.88***	(0.85,0.91)
Heart disease	0.85***	(0.82,0.88)	0.88***	(0.86,0.91)
Neurological conditions	0.82***	(0.80,0.84)	0.82***	(0.79,0.86)
Depression	0.65***	(0.62,0.67)	0.77***	(0.75,0.80)
Anxiety disorders	0.65***	(0.61 0.68)	0.86***	(0.81,0.90)
Substance use disorders	0.39***	(0.37,0.40)	0.64***	(0.62,0.66)
Women				
Cancer	0.85***	(0.82,0.88)	0.90***	(0.87,0.94)
Diabetes	0.78***	(0.76,0.81)	0.88***	(0.86,0.91)
Heart disease	0.84***	(0.81,0.87)	0.90***	(0.86,0.93)
Neurological conditions	0.81***	(0.79,0.82)	0.86***	(0.84,0.88)
Depression	0.63***	(0.61,0.64)	0.78***	(0.76,0.80)
Anxiety disorders	0.71***	(0.68,0.74)	0.87***	(0.83,0.90)
Substance use disorders	0.37***	(0.34,0.39)	0.60***	(0.57,0.63)

Abbreviations: *HR* hazard ratio, *CI* confidence interval

^a^ *P*-values ** < .01, *** < .001 Model adjusted for age, onset year of unemployment, native language, marital status, education, occupational status, number of individuals living in the same household, home ownership, region of residence, unemployment history, number of unemployment spells, binary measure for any remaining comorbidity, and for women, births over the two preceding years. All specific health conditions (cancer, diabetes, heart disease, neurological conditions, depression, anxiety disorders, substance use disorders) modelled simultaneously. Stable employment was defined as still being employed at the end of the calendar year following the initial date of employment, and unstable employment as no longer being employed. For the definitions of the specific health conditions, please see Measurements and Additional file 2

### Sensitivity analyses

Our results from the multivariate Cox proportional hazard models were robust to competing risks. The exclusion of short unemployment spells and the inclusion of those with loose attachments to the labor market produced similar results to those of our main analyses (Additional file 1).

## Discussion

This study examines health-related selection from unemployment into employment among Finnish residents aged 30 to 60. We assessed various aspects of health from physical to psychiatric as well as specific health conditions and addressed their importance in finding employment and in employment stability. We demonstrated that poor health was negatively associated with employment in a two-year follow-up. The most detrimental health problems for finding work were alcohol-related and psychiatric conditions. The former halved the likelihood and the latter reduced it by almost 30% among both men and women. Additionally, alcohol-related and psychiatric conditions were more strongly associated with finding stable than unstable employment. It thus appears that these conditions constitute a risk factor both for prolonged periods of unemployment as well as for unstable career trajectories.

### Health and employment among the unemployed: comparisons with previous studies

Few previous studies have investigated alcohol-related health in relation to employment among the unemployed, but we showedit to be an important risk factorfor employment, and in particular, for stable employment. A Norwegian study reported alcohol use disorders to be unassociated with 5-year employment among the long-term unemployed, but this is likely due to a lack of statistical power (*n* = 228), as none of the 18 subjects with alcohol use disorders became employed [9].

The prior evidence is more consistent for the association between psychiatric conditions and finding employment. In Norway, the chances of finding employment within two years was 70% lower (*p*-value < 0.001) for those unemployed with any psychiatric diagnosis [7]. Among the Danish unemployed, any psychiatric medication purchases were also negatively associated with employment (OR 0.46; 95% CI: 0.34, 0.63) [32]. These results are in line with our finding that the unemployed with any psychiatric condition, depression or anxiety disorders were approximately 30% less likely to be employed over the 2-year follow-up.

Previous research assessing employment in terms of physical health is rare and predominantly based on small samples that produce imprecise point estimates [7, 8]. Contrary to our results, two Norwegian studies reported no association between any physical conditions and finding employment (*N* = 277, 228). Conversely, purchasing any prescription medication related to a physical condition was demonstrated to be detrimental to employment among the Danish unemployed in 2006–2008 (*n* = 1806) [32]. Previous studies addressing specific physical health conditions are also rare. Our results for heart disease are congruent with a study conducted in Sweden, in which self-reported circulatory symptoms were negatively associated with employment among respondents aged 25–54 in 1992–1993 (*n* = 452) [10]. Altogether, our results based on a large sample and objective and consistent measurement of both unemployment and physical conditions demonstrates that physical health is predictive of transitions from unemployment to employment.

### Explaining health-related selection into employment

Two explanations are plausible for the health-related selection from unemployment into employment. First, employers may discriminate against applicants with health-related gaps in their employment history [42], no recent work experience [43], or those that display signs of poor health, especially if these cues point to alcohol-related or psychiatric problems [25, 26]. In support of this hypothesis, we demonstrated that alcohol-related and psychiatric conditions were particularly harmful to finding employment. It is unclear to what extent employment protection legislation in the Nordic welfare states hinders discrimination against applicants with health conditions in hiring [5, 24]. Second, poor health could make the unemployed individual less capable or motivated to seek employment, or it could limit the options available to them. Chronic conditions may hinder an individual’s education as well as building skills and experience, and thus limit employment prospects [14, 15]. This hypothesis was not supported by our results, as adjusting for education, occupational status and unemployment history did not explain the lower chances of becoming employed among those with poor health. It is therefore more likely that poor health may either narrow the available options down to positions with lower health-related demands (both physical and social), or that symptoms and health-related stress may hinder the job search [16–19].

Regarding the general discussion on health-related selection into and out of employment, our results, together with the literature, suggest differing selection processes for different aspects of health. For example, while hospitalizations related to cancer and ischemic heart disease [11] and circulatory symptoms [10] did not seem to increase the risk of job loss among employed Swedes, our results suggest that physical conditions do hinder moving from unemployment to employment. Physical health may thus be less associated with the risk of job loss (e.g., due to employee protection legislation), and more with finding new work [5]. Psychiatric and alcohol-related conditions on the other hand appear to be associated with both job loss [11] and finding employment. Taken together, individuals with long-standing health conditions – physical or psychiatric – are likely to require additional support following their job loss.

### Health-related selection into stable and unstable employment

Previous studies have hypothesized that poor health, especially alcohol-related and psychiatric conditions, may be a determinant of precarious employment [34]. We found some support for this hypothesis, as alcohol-related and psychiatric conditions were particularly harmful for stable employment. This indicates that these conditions may hinder both procuring employment as well as strong and stable attachments to the labor market. Prior studies have established that those with psychiatric conditions face discrimination when looking for work as well as during employment [44] and they also have a higher risk of job loss [11]. Some psychiatric conditions may also be episodic in nature, which translates to a time-varying capacity to work. Psychiatric health may therefore be particularly important for job loss and the retention of stable employment.

Our results also imply that health is likely to contribute to accumulating disadvantage [10]. Those with poor health who lose their employment may be in danger of lengthy unemployment spells and unstable career trajectories which, in addition to being costly to society, have been linked to individual level outcomes such as worsening labor market position [45] and subsequent poor health [1]. To support employment into stable jobs among all unemployed, we need to identify vulnerable population groups with special problems related to their workability [46, 47], to modify job demands to better accommodate people in poorer health, and to reduce health-related stigmatization of those unemployed with poor health. Additional studies are needed to identify the best practices for supporting swift and stable employment among those unemployed with health conditions.

### Strengths and weaknesses

The strengths of this study are the large-scale, high-quality longitudinal data that enabled us to separate specific health conditions and employment outcomes. The data also allowed us to form a measure of any alcohol-related conditions that includes both physical illness and alcohol use disorders, which is a notable strength because there is likely to be considerable overlap between the two. Another strength is that our unemployment and health measures include exact dates. In addition, the analysis does not suffer from problems related to population sub-group differences in reporting styles, or higher rates of loss to follow-up among those in poor health.

Our health measures capture a wide range of clinically relevant, chronic conditions. We utilize data from specialized health care, which includes hospitalizations, outpatient care and emergency care visits, as well as data on special reimbursement rights for certain physical conditions. The special reimbursement rights are registered irrespective of whether the patient purchases the medications and therefore do not reflect an adherence to treatment. However, the special reimbursement data only includes certain conditions, and this prevents us from identifying some mental health-related chronic conditions such as insomnia, or conditions that are primarily treated with pain medication. This study is also blind to untreated or less severe conditions that do not appear in the registers.

Similarly, we cannot identify those who do not register as unemployed (e.g., the discouraged unemployed, or those not financially dependent on unemployment benefits due to severance pay packages). Thus, those who do not register are likely either to be in an advantageous position (e.g., with regards to employment history or a better financial situation due to severance packages) or conversely, in an extremely disadvantaged position (discouraged workers) when compared to the registered unemployed. Inclusion of the discouraged workers would likely strengthen the association between health and employment. In contrast, inclusion of those on severance packages may weaken the association. This group is likely to be relatively healthy and affluent, with less financial pressure to find employment. Therefore, they are likely to experience longer unemployment spells [48]. However, little is known about the number of people who receive severance packages or the level of pay and benefits they receive, as these are considered private agreements with employers and employees. Overall, the magnitude and joint effects of these two tendencies are unclear, but they could potentially cancel each other out.

Finally, using register data, we cannot control for job attitudes such as motivation, willingness to relocate for work or to take up employment in a new field. The contribution of job attitudes to health-related selection therefore remains unknown.

## Conclusions

While there is a large body of literature on the causality and health-related selection in the association between unemployment and health, few studies have addressed the role of health in finding employment among the unemployed. We demonstrated that any alcohol-related conditions, psychiatric conditions, self-harm, injuries, and physical conditions were all adversely associated with employment among unemployed Finnish men and women. We also show that alcohol-related and psychiatric conditions may be associated with unstable career trajectories, as both appear to be stronger predictors of stable employment than unstable employment. These results contribute to the more general discussion on the role of causality and health-related selection in the association between unemployment and health, as we conclude that health-related selection into employment is likely to contribute to the poorer health observed among those who remain unemployed. These findings are also important from a policy perspective, as those unemployed who experience health problems may need additional support to enhance their swift and stable employment after their job loss.

## Supplementary Information

**Additional file 1**

**Additional file 2**

## Data Availability

Following the data protection regulations of the national register-holders, we cannot make the data available to third parties. Interested researchers may contact the following register-holding public institutions: Statistics Finland (http://www.stat.fi/tup/mikroaineistot/index_en.html), The Social Insurance Institution of Finland (http://www.kela.fi/web/en/research-data-requests).

## Abbreviations

CI: Confidence interval
HR: Hazard ratio
OR: Odds ratio
SD: Standard deviation
SE: Standard error
