# Supplementary material for: Health-related selection into employment among the unemployed
Source: BMC Public Health. 2022 Apr 5;22:657. doi: 10.1186/s12889-022-13023-0 (PMC8985275; doi:10.1186/s12889-022-13023-0)
Supplement: Supplementary file 1 — Additional file 1 [file 12889_2022_13023_MOESM1_ESM.pdf]

Additional file 1

Supplementary table 1 Sensitivity analyses, the association between different general aspects of health and finding stable and unstable employment among unemployed Finnish men and women aged 30–60 in 2009–2018[a]

	Men		Women	
	HR	95% CI	HR	95% CI
Unemployment episodes lasting less than 31 days excluded.				
Any alcohol-related conditions, poisonings	0.53	(0.52,0.55)	0.47	(0.46,0.49)
Any psychiatric conditions, self-harm	0.69	(0.67,0.70)	0.71	(0.70,0.72)
Any injuries	0.92	(0.91,0.93)	0.91	(0.90,0.92)
Any physical conditions	0.90	(0.90,0.91)	0.90	(0.90,0.91)
Observations	1,349,975		1,508,548	
All unemployment episodes included				
Any alcohol-related conditions, poisonings	0.50	(0.49,0.52)	0.46	(0.43,0.48)
Any psychiatric conditions, self-harm	0.71	(0.69,0.73)	0.70	(0.68,0.71)
Any injuries	0.89	(0.88,0.90)	0.88	(0.86,0.89)
Any physical conditions	0.91	(0.90,0.92)	0.91	(0.90,0.92)
Observations	1,507,547		1,947,314	

Abbrevations: HR, hazard ratio; CI, confidence interval.

[a] All p-values <.001. Model adjusted for age, onset year of unemployment, native language, marital status, education, occupational status, number of individuals living in the same household, home ownership, region of residence, unemployment history, number of the unemployment spell, and for women, births over the two preceding years. All general aspects of health measures modelled simultaneously. Stable employment was defined as still being employed at the end of the calendar

year following the initial date of employment, and unstable employment no longer being employed. For the definitions of the general aspects of health measures, please see Measures and Additional file 2.

Supplementary table 2 Sensitivity analyses, the association between specific health conditions and finding stable and unstable employment among unemployed Finnish men and women aged 30–60 in 2009–2018[a]

	Men		Women	
	HR	95% CI	HR	95% CI
Unemployment episodes lasting less than 31 days excluded.				
Cancer	0.90***	(0.86,0.93)	0.87***	(0.85,0.90)
Diabetes	0.85***	(0.83,0.87)	0.83***	(0.81,0.85)
Heart disease	0.87***	(0.85,0.89)	0.86***	(0.84,0.88)
Neurological conditions	0.82***	(0.80,0.84)	0.82***	(0.81,0.84)
Depression	0.70***	(0.68,0.72)	0.68***	(0.67,0.70)
Anxiety disorders	0.74***	(0.71,0.76)	0.77***	(0.75,0.79)
Substance use disorders	0.53***	(0.51,0.54)	0.48***	(0.46,0.50)
Observations	1,349,975		1,508,548	
All unemployment episodes included				
Cancer	0.93*	(0.87,1.00)	0.89***	(0.86,0.93)
Diabetes	0.88***	(0.85,0.92)	0.87***	(0.84,0.91)
Heart disease	0.88***	(0.85,0.92)	0.87***	(0.84,0.90)
Neurological conditions	0.83***	(0.80,0.86)	0.83***	(0.82,0.85)
Depression	0.71***	(0.68,0.73)	0.66***	(0.65,0.68)
Anxiety disorders	0.76***	(0.71,0.81)	0.76***	(0.73,0.79)

Substance use disorders	0.49***	(0.47,0.50)	0.46***	(0.43,0.49)
Observations	1,507,547		1,947,314	

Abbrevations: HR, hazard ratio; CI, confidence interval.

[a] P-values, *<.05, ** <.01, ***<.001. Model adjusted for age, onset year of unemployment, native language, marital status, education, occupational status, number of individuals living in the same household, home ownership, region of residence, unemployment history, number of unemployment spells, binary measure for any remaining comorbidity, and for women, births over the two preceding years. All specific health conditions (cancer, diabetes, heart disease, neurological conditions, depression, anxiety disorders, substance use disorders) modelled simultaneously. Stable employment was defined as still being employed at the end of the calendar year following the initial date of employment, and unstable employment as no longer being employed. For the definitions of the specific health conditions, please see Measurements and Additional file 2.

Competing risk regression

Supplementary table 3 Sensitivity analysis, the association between different general aspects of health and finding stable and unstable employment among unemployed Finnish men and women aged 30-60 in 2009-2018 from Fine and Gray models with competing risks (SHR)[a]

	Men		Women	
	SHR	95% CI	SHR	95% CI
Any employment				
Any alcohol-related conditions, poisonings	0.51***	(0.48,0.54)	0.46***	(0.43,0.51)
Any psychiatric conditions, self-harm	0.70***	(0.67,0.73)	0.70***	(0.67,0.72)
Any injuries	0.91***	(0.88,0.93)	0.91***	(0.88,0.93)
Any physical conditions	0.90***	(0.88,0.92)	0.90***	(0.89,0.91)
Stable employment				

Any alcohol-related conditions, poisonings	0.35***	(0.31,0.39)	0.37***	(0.32,0.43)
Any psychiatric conditions, self-harm	0.58***	(0.54,0.62)	0.64***	(0.61,0.67)
Any injuries	0.96*	(0.92,0.99)	0.89***	(0.86,0.93)
Any physical conditions	0.91***	(0.89,0.94)	0.90***	(0.88,0.92)
Unstable employment				
Any alcohol-related conditions, poisonings	0.70***	(0.65,0.75)	0.75***	(0.67,0.84)
Any psychiatric conditions, self-harm	0.85***	(0.80,0.91)	0.90***	(0.86,0.95)
Any injuries	0.88***	(0.85,0.92)	0.96	(0.92,1.01)
Any physical conditions	0.95***	(0.92,0.97)	0.98*	(0.95,1.00)
Observations	135,505		150,347	

Abbrevations: SHR, subdistribution hazard ratio; CI, confidence interval.

[a] P-values, *<.05, ** <.01, ***<.001. Model adjusted for age, onset year of unemployment, native language, marital status, education, occupational status, number of individuals living in the same household, home ownership, region of residence, unemployment history, number of the unemployment spell, and for women, births over the two preceding years. All general aspects of health measures modelled simultaneously. Stable employment was defined as still being employed at the end of the calendar year following the initial date of employment, and unstable employment no longer being employed. For the definitions of the general aspects of health measures, please see Measures and Additional file 2.

Competing risk regression accounts for the fact that the individual may experience other events that prevent employment over the two-year follow up. Given that for example death and retirement may prevent re-employment, we additionally conducted a Fine and Gray competing risk regressions for finding any, stable or unstable employment, with the competing risks of exiting unemployment for education or training, retirement, migration, or death. While the competing risk regression accounts

for the fact that the individual may experience other events such as death that prevent employment over the two-year follow up, the results depict relative changes in the employment rate among subjects who have not experienced employment but may have experienced a competing event and are therefore less readily interpretable as our main results derived from the Cox models. As these models are computationally intensive and our data are large, we conducted the estimates on a 10% random sample of our data.
