# Supplementary material for: Health-related selection into employment among the unemployed
Source: BMC Public Health. 2022 Apr 5;22:657. doi: 10.1186/s12889-022-13023-0 (PMC8985275; doi:10.1186/s12889-022-13023-0)
Supplement: Supplementary file 2 — Additional file 2 [file 12889_2022_13023_MOESM2_ESM.pdf]

Additional file 2 Categories of International Classification of Diseases Revisions 10 main diagnoses for hospitalization and Prescription Register's special reimbursement utilized in the study

Aspects of health, specific health conditions	Specialized health care visit diagnoses	Special reimbursement category
Any alcohol use-related conditions, poisonings	F10-F16, F18, F19, G312, G4051, G621, G721, I426, K292, K70, K852, K860, O354, P043, Q860, T40, X42, X45, F10-F19	
Any mental health, self-harm	F20-F69, X60-X84, Y87.0	
Any accidents, violence	T25-T29, T41-T65, V01-X41, X43, X44, X46-X59, Y10-Y15, Y85-Y86, W00-W99, X85-Y09, Y87.1	
Any physical conditions, excluding alcoholic diseases	A00-B99, C00-D48, E00-E90, I00-I425, I427-I99, J00-J64, J66-J99, K00-K93, L00-L99, M00-M99, N00-N99, P00-P99, Q00-Q99, R00-R94	
Cancer	C00-C97	Breast (115), prostatic (116), gynecological (128), other malignant tumors (130), melanoma, renal cancer (180)
Diabetes	E10–E14	Diabetes (103)
Neurological conditions	G00–G19, G20-G99	Parkinson's disease (110), epilepsy (111), multiple sclerosis (109), apoplectic symptoms (108), trigeminusneuralgia, glossofaryngikusneuralgia (119)
Heart disease	I00–I09, I20–I52	Cardiac insufficiency (201), coronary heart disease (206), arrhythmia (207)
